# Supplementary material for: Rapid turnover of life-cycle-related genes in the brown algae
Source: Genome Biol. 2019 Feb 14;20:35. doi: 10.1186/s13059-019-1630-6 (PMC6374913; doi:10.1186/s13059-019-1630-6)
Supplement: Supplementary file 3 — Protein alignments, corresponding Gblocks html file and CDS sequences for the lineage conserved gametophyte-biased genes. (ZIP 2109 kb) [file 13059_2019_1630_MOESM3_ESM.zip › Con_GA_alignments/Ec-21_002360.aa_ali.fasta-gb.htm]

Ec-21\_002360.aa.fasta.trans.aa\_ali.fasta


## Gblocks 0.91b Results

Processed file: **Ec-21\_002360.aa.fasta.trans.aa\_ali.fasta**  
Number of sequences: **2**  
Alignment assumed to be: **Protein**  
New number of positions: **412** (selected positions are underlined in blue)

```
                         10        20        30        40        50        60
                 =========+=========+=========+=========+=========+=========+
Ec-21_002360     ------------------------------------------------------------
MSTRG.21514.1    PSSTEHSRRLARCCWAAMPITLVGSSRELKNRRLLPPIDLLDLLGECNGPGAAAAMDEPR
                                                                             


                         70        80        90       100       110       120
                 =========+=========+=========+=========+=========+=========+
Ec-21_002360     ------------------------------------------------------------
MSTRG.21514.1    SPRDSSFPPRQAAFGAKSDRTRKPGEATNRSNVATTQSPRSLDTGDSYAEDMHPFMALLN
                                                                             


                        130       140       150       160       170       180
                 =========+=========+=========+=========+=========+=========+
Ec-21_002360     ------------------------------------------------------------
MSTRG.21514.1    GRQYPYIGGQESSSCTQQSTGRAALSVSGAAPTAAVPDITAVAEGAMSSALVSGEAARAA
                                                                             


                        190       200       210       220       230       240
                 =========+=========+=========+=========+=========+=========+
Ec-21_002360     ------------------------------------------------------------
MSTRG.21514.1    SENQADTLPPTPHPSAAKAENLAETSAPTASSDKTNAPKHTVGDEGRQRLGMEPSEADRN
                                                                             


                        250       260       270       280       290       300
                 =========+=========+=========+=========+=========+=========+
Ec-21_002360     -----------------------------------------MRKARGKSAAIPTS--SGD
MSTRG.21514.1    CDGDFLQGANLQAARPPAQPRPRRPPHYARPTSASKGVFNRRRRTRGPRLDIPTSSASGD
                                                           #############  ###


                        310       320       330       340       350       360
                 =========+=========+=========+=========+=========+=========+
Ec-21_002360     FANSLLRLCPALEARISDLRRDNTAAARFLSKAQATASEKARRPVGALVSRNRAVCALEG
MSTRG.21514.1    AVDSLLRLCPALEARMMDLRRDNTAAARFLHKAQVAAWQKARAKPGVLVSRNRAVCVLEG
                 ############################################################


                        370       380       390       400       410       420
                 =========+=========+=========+=========+=========+=========+
Ec-21_002360     AELSLLRREETATRAQRKREANRERRRRLERRRLATAEATRIKHEGPSLCEVRALEEAAL
MSTRG.21514.1    VELSLLRRQEAAVRSQHRRKANRERKRRMEVRRLAEAEAVRVKHEGPSLAELQAQEQALQ
                 ############################################################


                        430       440       450       460       470       480
                 =========+=========+=========+=========+=========+=========+
Ec-21_002360     LEKRQRFFMFAVVAIHSAIRWRNDWKERKWKMAKFAEALAGLAEQRQSEDVFEASVARVQ
MSTRG.21514.1    LERRQRFFMTVVIMINTTVLLRNQWKERMWKLAKFAEALVDVRAEESSPDIFEVSVKRIQ
                 ############################################################


                        490       500       510       520       530       540
                 =========+=========+=========+=========+=========+=========+
Ec-21_002360     RFVRRRQGAPGVAGGAAAAAGDRSGR--RNFIRCLKAWIPRAHGTWKRRRREGGVIIYDF
MSTRG.21514.1    RLFRHRQGHGSCCTWSGGGGGVTIGGREKNLIRRLRQWIPIAHARWKGRRREGGAVIHDF
                 ########                     ###############################


                        550       560       570       580       590       600
                 =========+=========+=========+=========+=========+=========+
Ec-21_002360     LKDARLSQNIKAIYRFRRKVMRCQVYVRGVSVVTRDRLLLLGLFFSRCEGRLRGRLAERV
MSTRG.21514.1    LKDARLTQNIKAIYRFRRKVIRCQIYVKGVRVITRDRLKLLGLFFARCEARLRERLAERV
                 ############################################################


                        610       620       630       640       650       660
                 =========+=========+=========+=========+=========+=========+
Ec-21_002360     RQQVQEEAAADLEIHRQLMRPSTSAAAA-----AAAAASSAIPA-----AAAGGNRWRRR
MSTRG.21514.1    RQQVLEEAAADLEVHRQLMRPSASAAATTVDKLAGTASVSPTPAGIACARGGGGGRWRSQ
                 ###########################      ###########        ########


                        670       680       690       700       710       720
                 =========+=========+=========+=========+=========+=========+
Ec-21_002360     TNKNGGD----AGDDDDGVSELMETVAALRLKSAQEELEMRTARPDVKHRLLEGLLGKVR
MSTRG.21514.1    QRCDSGSRRIGGSRDNDDVSKLMEAVAALRLKSALEELETRSAHPDVKRHLLQGLLEEVR
                 ######        ##############################################


                        730       740       750       760       770       780
                 =========+=========+=========+=========+=========+=========+
Ec-21_002360     AAHQSRMQLSSGGRGEDGGGGVGDARKGRGPGRNRRGVSLPEALFTVDDARELLTQRSPP
MSTRG.21514.1    TAHQGRIKLPK--KGC--------------------------------------------
                 #########                                                   


                        790       800       810       820       830       840
                 =========+=========+=========+=========+=========+=========+
Ec-21_002360     PSEGSASACDITVGLTKGNRASPNGSGELPGAPYSTVAVSRPGTGGGGGGSKSDFRPNTR
MSTRG.21514.1    ---------D--------GSAYEDGNAGFP-----------------PAG----------
                                     ##########                              


                        850       860       870
                 =========+=========+=========+==
Ec-21_002360     VPPMLLLKSVPEAVMCQLVLTAWEQSSSSGRQ
MSTRG.21514.1    --------------------------ARAGFR
```

```
Parameters used
Minimum Number Of Sequences For A Conserved Position: 2
Minimum Number Of Sequences For A Flanking Position: 2
Maximum Number Of Contiguous Nonconserved Positions: 8
Minimum Length Of A Block: 10
Allowed Gap Positions: None
Use Similarity Matrices: Yes
```

```
Flank positions of the 7 selected block(s)
Flanks: [283  295]  [298  488]  [510  627]  [634  644]  [653  666]  [675  729]  [801  810]  

New number of positions in Ec-21_002360.aa.fasta.trans.aa_ali.fasta-gb:  412  (47% of the original 872 positions)
```
